# Evaluation and Application of the Strand-Specific Protocol for Next-Generation Sequencing

**DOI:** 10.1155/2015/182389

**Published:** 2015-03-29

**Authors:** Kuo-Wang Tsai, Bill Chang, Cheng-Tsung Pan, Wei-Chen Lin, Ting-Wen Chen, Sung-Chou Li

**Affiliations:** ^1^Department of Medical Education and Research, Kaohsiung Veterans General Hospital, Kaohsiung, Taiwan; ^2^YourGene Biotechnology, Taipei, Taiwan; ^3^Institute of Bioinformatics and Systems Biology, National Chiao Tung University, Taiwan; ^4^Department of Parasitology, National Cheng Kung University Medical College, Tainan, Taiwan; ^5^Molecular Medicine Research Center, Chang Gung University, Taoyuan, Taiwan; ^6^Bioinformatics Center, Chang Gung University, Taoyuan, Taiwan; ^7^Genomics and Proteomics Core Laboratory, Department of Medical Research, Kaohsiung Chang Gung Memorial Hospital and Chang Gung University College of Medicine, Kaohsiung, Taiwan

## Abstract

Next-generation sequencing (NGS) has become a powerful sequencing tool, applied in a wide range of biological studies. However, the traditional sample preparation protocol for NGS is non-strand-specific (NSS), leading to biased estimates of expression for transcripts overlapped at the antisense strand. Strand-specific (SS) protocols have recently been developed. In this study, we prepared the same RNA sample by using the SS and NSS protocols, followed by sequencing with Illumina HiSeq platform. Using real-time quantitative PCR as a standard, we first proved that the SS protocol more precisely estimates gene expressions compared with the NSS protocol, particularly for those overlapped at the antisense strand. In addition, we also showed that the sequence reads from the SS protocol are comparable with those from conventional NSS protocols in many aspects. Finally, we also mapped a fraction of sequence reads back to the antisense strand of the known genes, originally without annotated genes located. Using sequence assembly and PCR validation, we succeeded in identifying and characterizing the novel antisense genes. Our results show that the SS protocol performs more accurately than the traditional NSS protocol and can be applied in future studies.

## 1. Introduction

Biological studies typically require sequencing mRNA and genomic DNA. Sanger dideoxy sequencing has been widely applied for decades [1, 2] since its invention. Although traditional Sanger dideoxy sequencing meets the demands of most sequencing jobs, it costs considerable time and money, which is less than satisfaction. The recent invention of next-generation sequencing (NGS) technologies provides alternatives for sequencing jobs [3–5]. NGS produces abundant sequence data in a short time, making it more economical and popular than the traditional Sanger dideoxy sequencing [6]. Therefore, NGS has become a powerful research tool, applied in a broad range of biomedical studies [7–12], including determining gene expression profiles.

Before sequencing with NGS technology, RNA samples should first be prepared using a standard protocol, by which the single-strand RNAs are first converted into double-strand complementary DNAs (cDNAs), followed by adaptor ligation and PCR amplification. The amplified cDNAs are then loaded onto a sequencing platform and then sequenced [6]. The generated sequence fragments are typically called sequence reads. When used to determine gene expression profiles, the generated sequence reads are mapped back to transcripts. And numerous tools are suitable for the mapping jobs [13–17]. For each transcript, by tabulating the number of reads mapped and by normalizing with sample size and transcript length, we can calculate the expression level of transcripts and genes in the unit of reads per kilo base per million mapped reads (RPKM).

However, the conventional sample preparation protocol eliminates strand specificity, resulting in collecting both the sense strand and the antisense strand of the original mRNA, without knowing which strand of the cDNA is the original mRNA. Therefore, when mapped back to transcripts, the reads mapped to the sense strand and antisense strand of transcripts are equally summarized without discrimination. Tabulating the read count in this manner seems applicable for most transcripts, but not for all.

Different genes can be located at the same genomic locus, overlapping each other. Such overlapping architectures are beneficial for gene regulation [18] and are thus conserved in the course of evolution [19]. The overlapping genes have been classified into different subgroups based on their orientations and overlapped proportions [20]. In this study, we are particularly interested in the overlapping genes at antisense strands. According to refFlat table (UCSC hg19), 2,536 human genes (4,424 transcripts) overlap at the antisense strand [21]. We call these “mutually overlapped genes” (mutually overlapped transcripts) in this study. In that case, the sequence reads mapped to both of the mutually overlapped transcripts are usually equally distributed to the two transcripts. However, distributing read count in this manner assumes that the two transcripts have equal expression abundance. Thus, distributing sequence reads in that way may cause bias.

Strand-specific protocols have recently been developed to overcome the drawbacks of conventional non-strand-specific protocols [22]. A previous study showed that the expression profile determined by strand-specific protocol was highly correlated with the ones by quantitative PCR and tilling array, providing high-quality sequencing data and holding the strand specificity [23]. Using the strand-specific protocols retains strand-specificity, such that we can map the sequence reads back to the exact strand of chromosome.

In this study, we focused on the comparison between SS and NSS protocols. We individually prepared the same RNA samples with SS and NSS protocols and then sequenced the samples using Illumina HiSeq platform. Using real-time quantitative PCR as a standard, we compared the gene expression profiles determined by SS and NSS protocols. Our result showed that the SS protocol is more unbiased in detecting gene expression levels for mutually overlapped transcripts. Further comparisons also showed that the sequence reads from SS protocols were comparable with the ones from other NSS protocols in many aspects. In addition, the SS protocol can be applied in identifying novel genes hidden at the antisense strand of known genes.

## 2. Materials and Methods

### 2.1. Gastric Cancer Cell Line and RNA

We extracted RNA samples from gastric cancer cells for sequencing, PCR, and qPCR. Five human gastric cancer cell lines (AGS, AZ-521, HR, NUGC, and TSGH) were obtained from the American Type Culture Collection and maintained in Dulbecco's modified Eagle's medium supplemented with 10% inactivated FBS (Invitrogen, Carlsbad, CA, USA). Total RNA of gastric cancer cells was extracted with TRIZOL (Invitrogen, Carlsbad, CA, USA) according to the manufacturer's protocol. Total RNA was treated with DNase I at 37°C for 30 min; then DNase I was removed with phenol-chloroform extraction and total RNA was reprecipitated using sodium acetate and ethanol. The RNA samples from AGS cell line were subject to NGS sample preparation protocols, including strand-specific and non-strand-specific, followed by sequencing with Illumina HiSeq platform. The protocol for strand-specific sample preparation is ScriptSeq v2 RNA-Seq Library Preparation Kit (Epicentre, USA). The protocol for non-strand-specific sample preparation is TruSeq RNA Sample Preparation Kit v2 (Illumina, USA).

### 2.2. Mapping the Sequence Reads Back to Transcripts

We mapped the NGS sequence reads back to transcripts to classify the sequence reads, mappable or unmappable, and to determine gene expression profiles. The mapping jobs were done with Bowtie [15] with a “-v3” parameter specified to allow three mismatches in the alignments. When mapping the reads form the strand-specific protocol, the “–norc” parameter was specified to allow only the sense strand of transcripts was aligned.

### 2.3. Identification of Novel Transcripts

The collected strand-specific sequence reads were assembled with Trinity [24] with the default parameters, generating the sequences of isotigs. Then, the isotigs were mapped back to human genome (hg19) with blat [25]. Only the blat matches no less than 95% identity were considered. We further examined the blat alignment results to demand that the isotigs defined to have multiple exons should follow the GT-AG rule at their introns.

### 2.4. Reverse Transcription Real-Time PCR

2 ug of total RNA (DNase I treatment) was reverse-transcribed with oligo (dT)_15_ primers and SuperScript III Reverse Transcriptase according to the user's manual (Invitrogen, Carlsbad, CA, USA). The reaction was performed with incubation at 42°C for 1 hr; then, the enzyme was subsequently inactivated by incubation at 85°C for 5 min. The cDNA was used for the real-time PCR analysis with gene specific primers and gene expression was detected using a SYBR Green I assay (Applied Biosystems, Foster City, CA, USA). The expression levels of antisense RNA (TR001 and TR002) were examined as follows: 94°C for 10 min, followed by 35 cycles of 94°C/1 min, 60°C/1 min, and 72°C/30 s, with a final extension at 72°C for 10 min using a PCR thermocycler and HotStart* Taq* DNA polymerase (Qiagen, Hilden, Germany). The PCR products were separated on 2% agarose gel; then PCR products were extracted from agarose gel using gel extraction kit (Qiagen, Hilden, Germany), and PCR products were subjected for sequencing. The primer sequences used in this study are listed in Supplementary Table  1 (see Supplementary Material available online at http://dx.doi.org/10.1155/2015/182389).

## 3. Results and Discussion

### 3.1. Mutually Overlapped Transcripts at the Antisense Strands

The UCSC Genome Bioinformatics mapped the transcript sequences back to genomes, defining the exact genomic boundaries of transcript exons. Such genomic locus information was recorded in the refFlat table. However, a fraction of the transcripts exhibited multiple-genomic loci. In this study, we only considered the transcripts with one genomic locus. Consequently, we analyzed the genomic coordinate information of 39,566 transcripts (from 23,152 genes).

**Figure 1:**
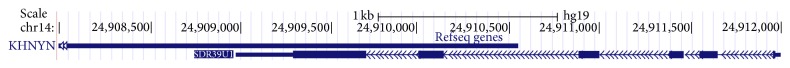
The details of the two overlapped genes. The transcript NM_015299, encoded by KHNYN, has eight exons, while NM_020195, encoded by SDR39U1, has 6 exons. NM_015299 and NM_020195 are located at the plus and minus strands of chromosome 14, respectively. The eighth exon of NM_015299 is overlapped by the fifth and sixth exons of NM_020195. The rest of exons of NM_015299 are ignored for simplicity.

For the one-locus transcripts, by comparing the genomic coordinates we examined whether they were mutually overlapped by other transcripts at the antisense strand. As a result, 4,424 transcripts (from 2,536 genes) are mutually overlapped with other transcripts.  Figure 1 demonstrates a pair of such overlapped transcripts. NM_015299 (the transcription product of* KHNYN*, located at the plus strand of chromosome 14) and NM_020195 (the transcription product of* SDR39U1*, located at the minus strand of chromosome 14) mutually overlap each other at their antisense strands. The eighth exon of NM_015299 is overlapped by the fifth and sixth exons of NM_020195, leading to the ambiguous distribution of sequence reads.

When researchers determine gene expression profiles by calculating RPKM values, the NSS sequence reads mapped back to the overlapped regions (e.g., exons 5 and 6 of NM_020195) are typically equally distributed to the overlapped transcripts. However, distributing reads in that manner assumes that the overlapped transcripts have equal expression abundances, which could lead to a biased estimation.

### 3.2. Expression Correlation between Strand-Specific and Non-Strand-Specific Protocols

To compare the SS protocol with the NSS protocol in determining gene expression profiles, we extracted and prepared RNAs from AGS cells with the SS and NSS sample preparation protocols. We further sequenced the collected RNA samples with the Illumina HiSeq platform to generate two libraries of transcriptome sequence reads. As a result, 85.5 (NSS) and 84.7 (SS) millions of reads with 101 bases in length were collected.

We then determined the RPKM values of the transcripts by mapping the sequence reads of SS and NSS libraries back to the transcripts, followed by calculating the correlation coefficient of RPKM values of the two libraries (see Section 2). In addition, for the overlapped transcripts, we evaluated the degree of overlapping by defining the “overlap” value: the proportion of the overlapped length to the initial length. Therefore, for NM_015299, the overlap value is 13.84%, whereas, for NM_020195, the overlapped fraction is up to 70.76% (Figure 1).

**Figure 2:**
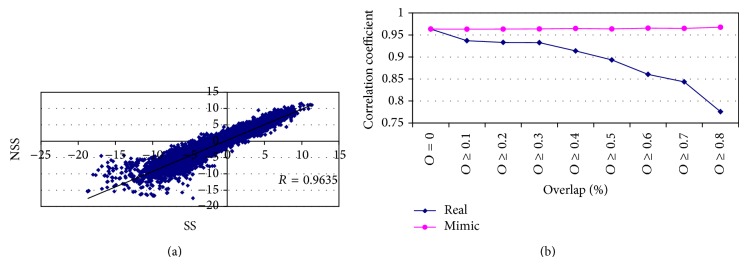
The expression correlation of transcripts between SS protocol and NSS protocol. The RPKM values of nonoverlapped transcripts were transformed with logarithm function with base 2. To avoid extreme values, only the transcripts with read count larger than 0.0001 in both libraries were considered. (a) The scatter plot of RPKM values derived from the SS and NSS libraries. The expression profile of nonoverlapped transcripts derived by SS protocol is highly correlated with the one by NSS protocol. (b) Based on the overlap percentage, the overlapped transcripts were classified into sets and the correlation coefficients were calculated in the sets (real). According to the sizes of different overlap sets (Supplementary Table  2), we resampled the nonoverlapped transcripts to mimic the correlation coefficient of overlapped sets. By repeating 100 times, the average values of correlation coefficient were drawn (mimic).

When the mutually overlapped transcripts are excluded, the expression profiles determined by the SS and NSS protocols are highly correlated (with a Pearson's correlation coefficient 0.9635, Figure 2(a)). However, when the overlapped transcripts are isolated into sets based on the overlap percentage, the expression profiles determined by the two protocols become less and less correlated with the increase of the overlap percentage (Figure 2(b), real). The detailed information of sets is available in Supplementary Table  2. For an unbiased comparison, we resampled the transcripts in *O* = 0 set to mimic the overlap sets. The result shows consistent correlation coefficient among sets (mimic). Therefore, the divergence between sets (real) was not caused by the difference on set size. In summary, an increased overlapping proportion causes a higher divergence between the SS and NSS libraries. So, we wonder which protocol more precisely determines the expression profiles of the overlapped transcripts.

### 3.3. Real-Time PCR Evaluation of Expression Profiles

Because of the divergence between the SS and NSS protocols, we used real-time PCR as a reference to examine which protocol is less biased in determining expression profiles of the overlapped transcripts. We selected nine overlapped genes for examination as well as three nonoverlapped genes as the internal controls [26, 27]. The nine genes were randomly selected based on the criteria, 2.0 RPKM fold change between SS and NSS. However, COX11 is an exception owing to our interest in its regulation in gastric cancer cell lines. For the nine examined overlapped genes, we detected their Ct values, followed by deriving the abundance fold changes relative to the three internal controls. By three replications, we collected 81 abundance fold changes from Ct values (9 genes ×3 internal controls ×3 replicates) and also derived the abundance fold changes from RPKM values of SS and NSS protocols. The Ct and RPKM values of the 12 genes are available in Table 1.

**Figure 3:**
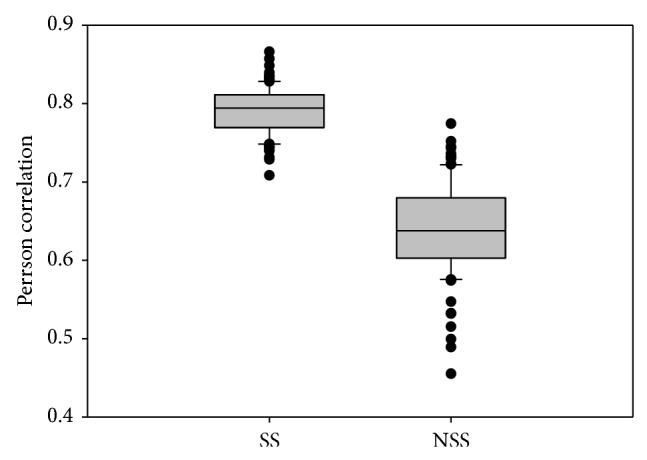
The real-time PCR evaluation of SS and NSS protocol. We used real-time PCR as standard to examine which protocol is less biased in determining expression profile of overlapped transcripts. After deriving the fold changes from Ct, SS RPKM and NSS RPKM, 81 values of fold change were collected. For a less biased result, we used bootstrap resampling for 100 times.

We subsequently calculated the correlation coefficients between Ct fold changes and SS RPKM fold changes as well as the ones between Ct fold changes and NSS RPKM fold changes. There are 4,424 overlapped transcripts, encoded by 2,536 genes. The comparison result from only nine genes could be biased. To eliminate the bias, we did bootstrap resampling for 100 times to calculate the correlation coefficients.  Figure 3 demonstrates that the Ct fold changes are higher correlated with the SS RPKM fold changes compared to the NSS RPKM ones (*P* value 1.41*E* − 50). In summary, using real-time PCR as a reference, we proved that the SS protocol is less biased than the NSS protocol in preparing the RNA samples and determining the expression levels of the overlapped genes.

In addition to the mutually overlapped transcripts, the sequence reads are usually mapped to multiple nonoverlapping transcripts, resulting in ambiguous mapped loci. These multiple loci sequence reads could be discarded without consideration in several studies. Or they could be distributed with statistical models. Actually, various statistics models have been invented for precise distribution of reads and for less biased estimation of gene expression profiles [28]. However, for the genes mutually overlapped at the antisense strands, using SS protocol can be a direct solution to this issue.

### 3.4. Examining the Performance and Strand Specificity

Although the SS protocol is less biased in determining gene expression profile than the NSS one, we did more comparisons between the SS and NSS libraries. We first evaluated them with quality score. Using FASTX-toolkit, we calculated the quality score of the sequence reads from the two libraries. Supplementary Figures  1 and 2 show that the two libraries have pretty similar patterns of score distribution across read position. The front-end bases of sequence read tend to have higher quality score than the rear-end ones, which is consistent with other NGS data. Moreover, the first several bases tend to have lower quality score.

**Figure 4:**
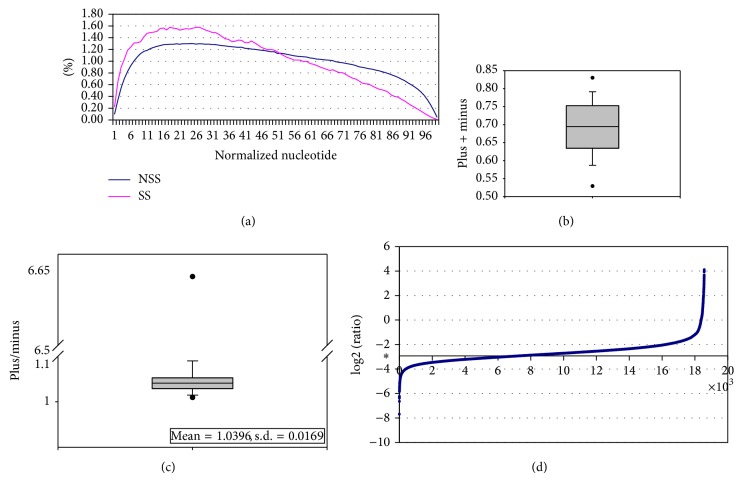
The evaluation of the performance and strand specificity. (a) The comparison of coverage between SS and NSS protocol. The lengths of all transcripts were normalized to 100 bases.* Y* axis denotes coverage frequency normalized with the number of all mapped bases. (b)* Y* axis denotes the mappable rate. For all libraries, including the SS library, the reads located at the sense and antisense strands are summarized. (c)* Y* axis denotes the sense strand mappable rate divided by the antisense strand one. (d)* Y* axis denotes the logarithm (base 2) values of ratios of the sense strand read count to the antisense strand one. ∗ at* Y* axis denotes −2.9357, indicating that 13.07% of the mappable reads from the SS library are located at the antisense strand of the transcripts. To eliminate dramatic values owing to ultimately small read count, only the nonoverlapped transcripts with read count ≥10 (18,573) at both strands were analyzed.

We then evaluated the protocols with the coverage across the transcripts. We tabulated the coverage frequencies of each base in each transcript, followed by normalizing the transcript length to 100 bases.  Figure 4(a) shows that the front end of transcripts tends to have higher coverage than the rear end, which is consistent with other studies [23]. In addition, compared with the NSS protocol, the front half of transcripts from the SS protocol have slightly higher coverage, whereas the rear half of transcripts from the SS protocol has slightly lower coverage.

We further evaluated the performance of the SS protocol in terms of mappable rate. For this purpose, we downloaded 16 NSS transcriptome libraries with accession number from ERR03088 to ERR030903 from NCBI SRA. Under the same parameters, we mapped these transcriptome sequence reads of 18 libraries (1 SS library, 1 NSS library, and 16 downloaded NSS libraries) back to human transcripts (refFlat hg19) with Bowtie [15]. As shown in Figure 4(b), the mappable rates of most libraries range from 65% to 75% and the two outliers belong to the downloaded SRA libraries. Such result shows that our libraries are comparable with others in terms of mappable rate.

We further examined whether strand specificity holds in the SS library. We specified the parameters “–norc" and “–nofw" as having only the sense strand and only the antisense strand of transcripts aligned, respectively. Since most libraries are non-strand-specific, the ratios of sense mappable rate to antisense mappable rate should be approximately one, which is proved by Figure 4(c). The outlier with a 6.65 value belongs to the SS library, where the sense and antisense mappable rates are 61.81% and 9.29%, respectively.

Since 13.07% of the mappable reads from the SS library are located at the antisense strand of the transcripts, we wonder if such antisense reads are generated by chance. In other words, they derive from an incomplete sample preparation protocol, losing strand specificity. Or there could be novel genes hidden at the antisense strand of known genes such as the case in Figure 1. Therefore, for the nonoverlapped transcripts, we tabulated the ratios of read counts at the antisense strand to those at the sense strand.  Figure 4(d) demonstrates that such ratio is equally distributed in most transcripts, with pretty low values. So, the reads at the antisense strands of most transcripts can be viewed as non-strand-specific noises. However, the ratio may reach up to more than one in a small fraction of nonoverlapped transcripts. Such result demonstrates that the reads at the antisense strand are not non-strand-specific noises, which strongly implies that there could be novel genes hidden at the antisense strand of the known nonoverlapped transcripts.

### 3.5. Identification and Characterization of Novel Genes at the Antisense Strand

To exam whether there is any novel transcript hidden at the antisense strand of the known genes, we had the reads unmappable to the sense strand of the known transcripts assembled with Trinity [24] (Section 2). The assembled isotigs were further mapped to the human genome (hg19) with blat [25] (Section 2). By doing so, we identified 3,997 novel transcripts hidden at the antisense strands of 1,577 known nonoverlapped genes. These novel and known transcripts form new cases of mutually overlapped transcripts as illustrated in Figure 1.

**Figure 5:**
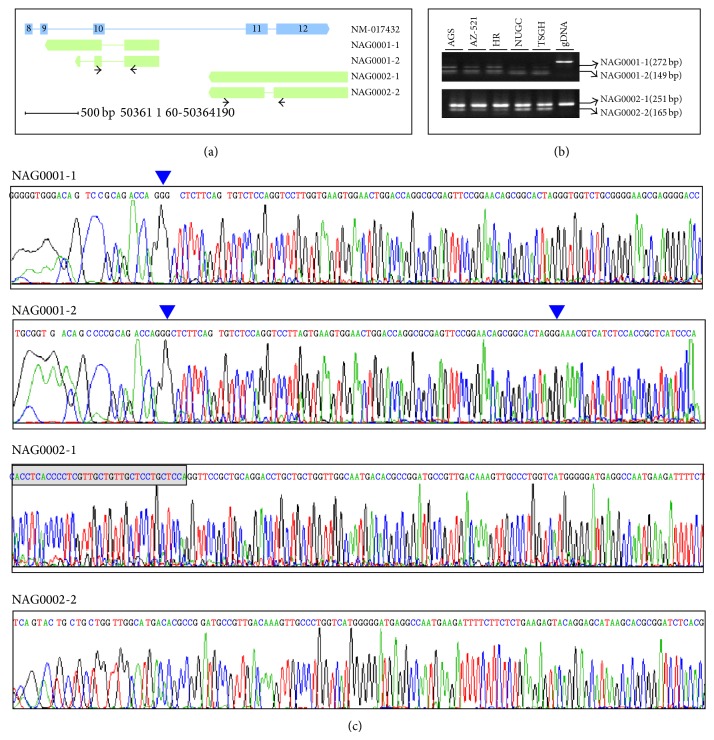
Illustration of novel antisense transcripts. (a) The thick and thin bars denote exon and intron, respectively. The terminal arrows denote the orientations of genes in chromosome. Right-ward arrow and left-ward arrow denote plus and minus strand, respectively. The blue and green bars denote known transcript and novel-antisense transcripts. The black thin arrows denote the positions of PCR primers. (b) NAG0001-1 and NAG0001-2 were detected with the same primer set. NAG0002-1 and NAG0002-2 were detected with the same primer set. We designed the PCR primers to avoid amplifying the antisense known transcripts. (c) The cloning and sequencing confirm the PCR results. The blue arrows mark the exon-exon junctions.

Figure 5(a) demonstrates two cases of such novel antisense genes. There are four transcripts identified at the antisense strand of NM_017432 (transcription product of PTOV-1), forming two independent genes, NAG0001 and NAG0002, where NAG denoted novel antisense gene. Like common protein-coding genes, the novel antisense genes also display complicated alternative splicing patterns. NAG0001 encodes two alternative splicing transcripts, which are entirely enclosed by NM_017432, spanning from exon 9 to intron 10 of NM_017432. Two alternative splicing transcripts of NAG0002, however, partially overlap the intergenic region, spanning from intron 10 to exon 12 of NM_017432. Supplementary Figure  3 illustrates more cases of the novel antisense genes. The detailed genomic information of the novel antisense transcripts is shown in Table 2.

To obtain experimental evidence, we selected seven novel antisense transcripts for PCR assay to validate the expression (Section 2).  Figure 5(b) and Supplementary Figure  3 show that we succeeded in detecting all seven transcripts in the gastric cell lines. Further cloning and sequencing confirmed the PCR result (Figure 5(c), Supplementary Figure  3). Our result proves that the SS protocol can be applied to identify novel antisense genes, which cannot be achieved with the NSS protocol.

Since the transcripts obtained from assembly alignments are usually partial lengths of the whole-length primary transcripts, we do not collect the complete exon-intron structures of novel antisense transcripts. Actually, the complete exon-intron structures of novel transcripts depend on the RACE experiment [29]. In this study, the two independent genes, NAG0001 and NAG0002, are less than 500 bases far from each other (Figure 5(a)). So we wonder whether they belong to the same gene but are assembled into two ones owing to the lack of the reads crossing the gap. If so, the two independent genes should have similar RPKM values compared with other novel genes.

To examine this hypothesis, we compared the fold changes of RPKM between novel antisense genes. As shown in Supplementary Figure  4, novel antisense genes a, b, and c are overlapped by the same known gene A, while d and e are overlapped by another known gene B. For the novel genes overlapped by the same known gene, we collected the fold changes of RPKM (e.g., FCab), forming the FCsame set. For the novel genes overlapped by different known gene, we also collected the fold changes of RPKM (e.g., FCad), forming the FCdiff set. We then compared the FC values of the FCsame and FCdiff sets. It turns out that the FC values of the FCsame set are significantly smaller than the ones of the FCdiff set (*t*-test *P* value < 2.2*E* − 16). Therefore, the novel genes overlapped by the same gene have similar expression levels, implying that they originated from the same gene but assembled into two ones owing to the lack of the reads crossing the gap.

Although we succeeded in detecting the expected transcripts with PCR assays, Figure 5(b) and Supplementary Figure  3 illustrate unexpected transcripts. As we know, we do not collect the complete collection of transcriptome. Therefore, there can be other alternative splicing isoforms detected with PCR but not identified with transcriptome assembly, which highlights that fact that alternative splicing patterns are much more complicated than we expected [7].

## 4. Conclusion

In this study, we first observed that known genes are located at the antisense strand of the same genomic loci, mutually overlapping each other. We then had the same RNA samples prepared with SS and NSS protocols. Using real-time quantitative PCR as a standard, we proved that SS protocol can provide less biased estimates of gene expression profile. In addition, SS protocol can be applied to identify the novel antisense genes. These findings together highlight the advantages of the strand-specific protocol for next-generation sequencing.

## Supplementary Material

In this study, we compared the performances of strand-specific (SS) and non-strand-specific (NSS) sample preparation protocols. We also investigated possible applications of SS protocols. For detailed illustrations, we provided this Supplementary Materials, including figures and tables.

## Figures and Tables

**Table 1:** The qPCR and RPKM results of the examined genes. We used qPCR to examine the expressions of nine overlapped genes and three nonoverlapped genes. EEF1A1 and HPRT1 are two commonly used internal control genes, while TMEM66 is the new internal control gene in this study. RPKM_SS and RPKM_NSS denote the RPKM values determined by the SS and NSS protocols, respectively. Ct1, Ct2, and Ct3 denote the Ct values from three independent qPCR experiments.

Gene	RPKM_SS	RPKM_NSS	Ct1	Ct2	Ct3
AMDHD2	3.58544	8.16441	26.30301	26.51864	26.41083
ATP6V1C2	2.31219	32.661	32.18005	31.57895	31.8795
COX11	23.49104	32.56102	21.27819	21.27837	21.27828
DALRD3	5.24003	17.97184	23.26547	23.24969	23.25758
MXD3	1.509837	8.58014	36.06333	35.29247	35.6779
POLR2I	8.01706	25.9707	19.39868	19.42806	19.41337
SDR39U1	6.96971	20.5453	23.02526	23.05474	23.04
SYNC	1.306066	5.69769	28.14738	28.05567	28.10153
TMOD1	1.30033	4.32226	27.98307	27.84805	27.91556
EEF1A1	2248.53	2270.11	13.11139	13.15321	13.13354
HPRT1	28.0151	28.3254	20.88358	21.07696	20.98027
TMEM66	126.355	126.437	18.85524	18.85882	18.85703

**Table 2:** The detailed genomic information of novel antisense transcripts. The genomic information is presented with the format of refFlat of UCSC.

Transcript	Chromosome	Strand	txStart	txEnd	exonStarts	exonEnds	Read count
NAG0001-1	chr19	−	50361371	50362420	50361371, 50362099	50361883, 50362420	180
NAG0001-2	chr19	−	50361659	50362420	50361659, 50361810, 50362099	50361684, 50361883, 50362420	34
NAG0002-1	chr19	−	50362914	50364190	50362914,	50364190,	121
NAG0002-2	chr19	−	50362914	50364190	50362914, 50363498	50363413, 50364190,	125.5
NAG0003-1	chr5	+	477198	477618	477198,	477618,	90.5
NAG0003-2	chr5	+	477198	478103	477198, 477920,	477580, 478103,	102.5
NAG0004-1	chr2	−	113341899	113342061	113341899,	113342061,	5
NAG0004-2	chr2	−	113342043	113342358	113342043, 113342247,	113342090, 113342358,	17
NAG0005-1	chr2	−	75157511	75186055	75157511, 75185858,	75158170, 75186055,	223
