# Supplementary material for: Evaluation and Application of the Strand-Specific Protocol for Next-Generation Sequencing
Source: Biomed Res Int. 2015 Mar 29;2015:182389. doi: 10.1155/2015/182389 (PMC4393923; doi:10.1155/2015/182389)
Supplement: Supplementary file 1 — In this study, we compared the performances of strand-specific (SS) and non-strand-specific (NSS) sample preparation protocols. We also investigated possible applications of SS protocols. For detailed illustrations, we provided this Supplementary Materials, including figures and tables. [file 182389.f1.doc]

**Supplementary Materials**

In this study, we compared the performances of strand-specific (SS) and non-strand-specific (NSS) sample preparation protocols. We also investigated possible applications of SS protocols. For detailed illustrations, we provided this S**upplementary Materials**, including figures and tables.

Supplementary Figure 1 page 2

Supplementary Figure 2 page 3

Supplementary Figure 3 page 4

Supplementary Figure 4 page 5

Supplementary Figure 5 page 6

Supplementary Table 1 page 7

Supplementary Table 2 page 9

**Supplementary Figure 1. The quality score of sequence reads from the NSS protocol.** This plot was made using FASTX-toolkit.


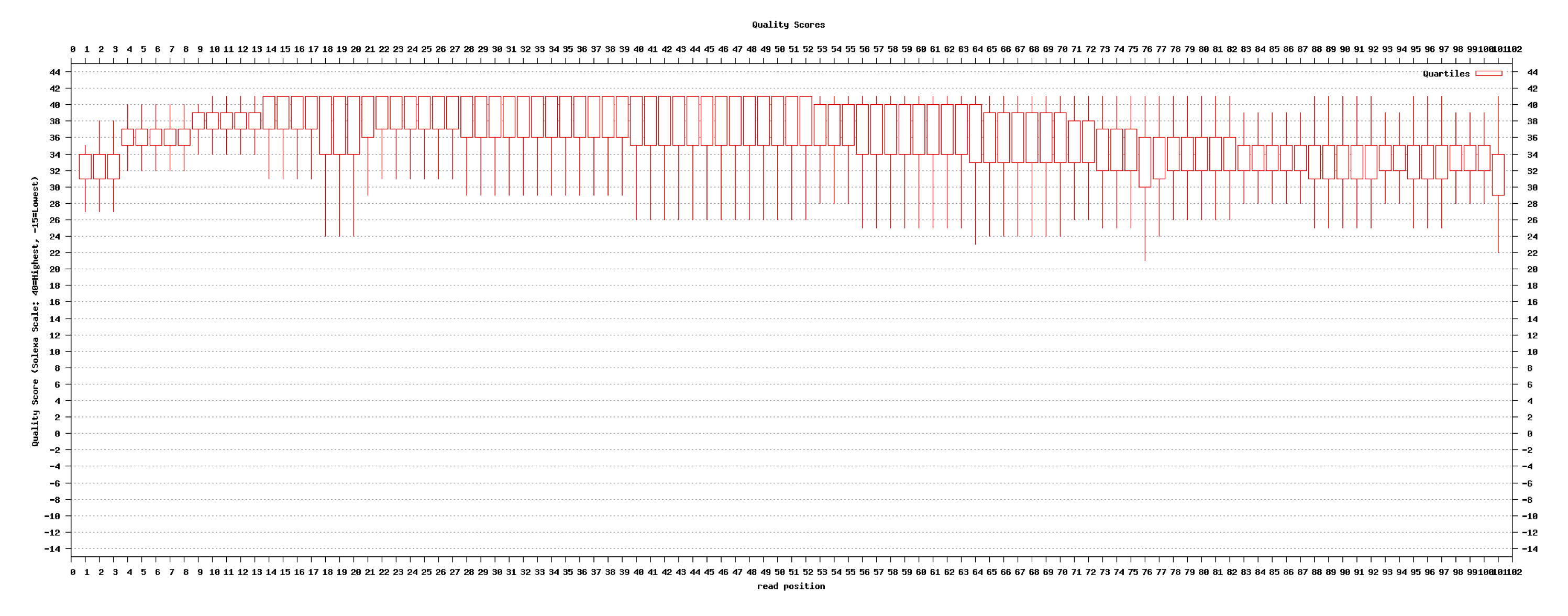


**Supplementary Figure 2. The quality score of sequence reads from the SS protocol.** This plot was made using fastx-toolkit.


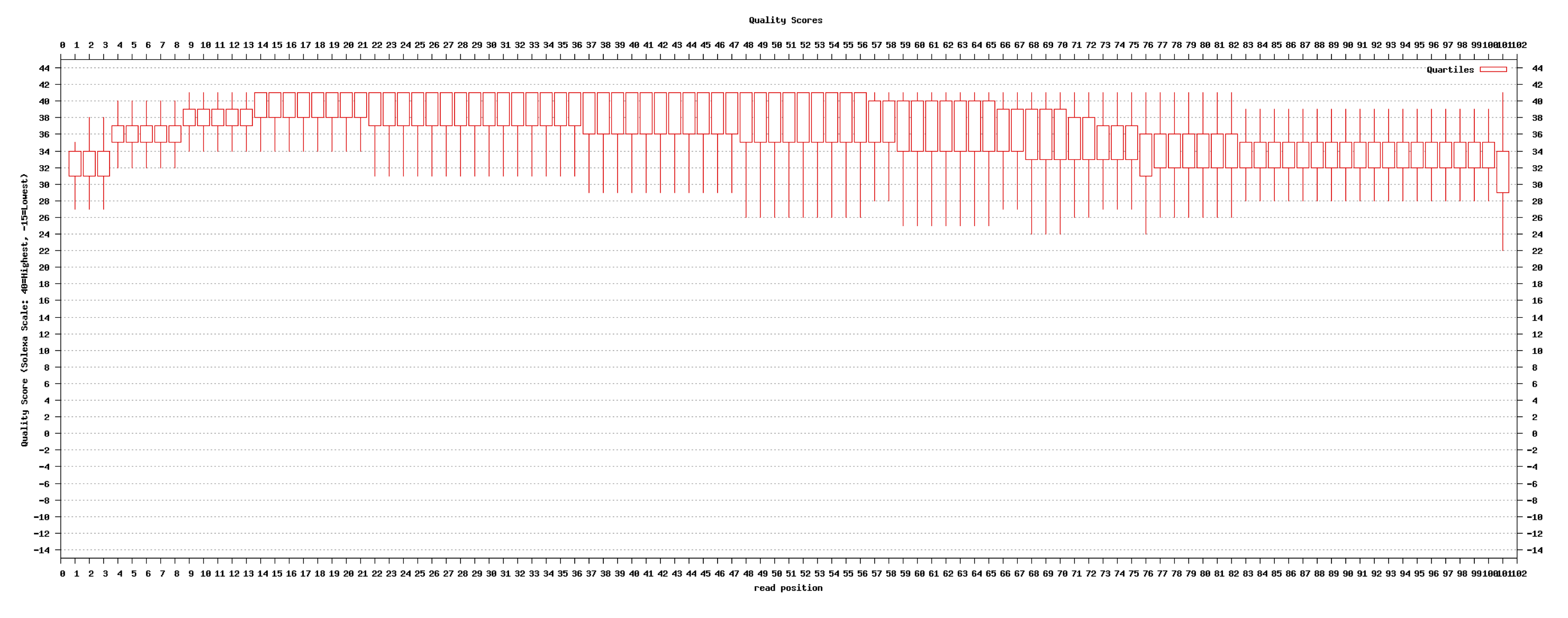


**Supplementary Figure 3. Illustration of other novel anti-sense transcripts.** More novel anti-sense transcripts are presented. (a) For simplicity, the intron length of NAG0005-1 was shorted to 1.669K from the original 27.69k. The black thin arrows denote the positions of PCR primers. (b) We designed PCR primers spanning the intron. So, only the transcripts (NAG0003-2, NAG-0004-2 and NAG0005-1) with intron were detected. Such design is to avoid amplifying the known transcripts at the anti-sense strand. (c) The cloning and sequencing confirm the PCR results. The blue arrows mark the exon-exon junctions.


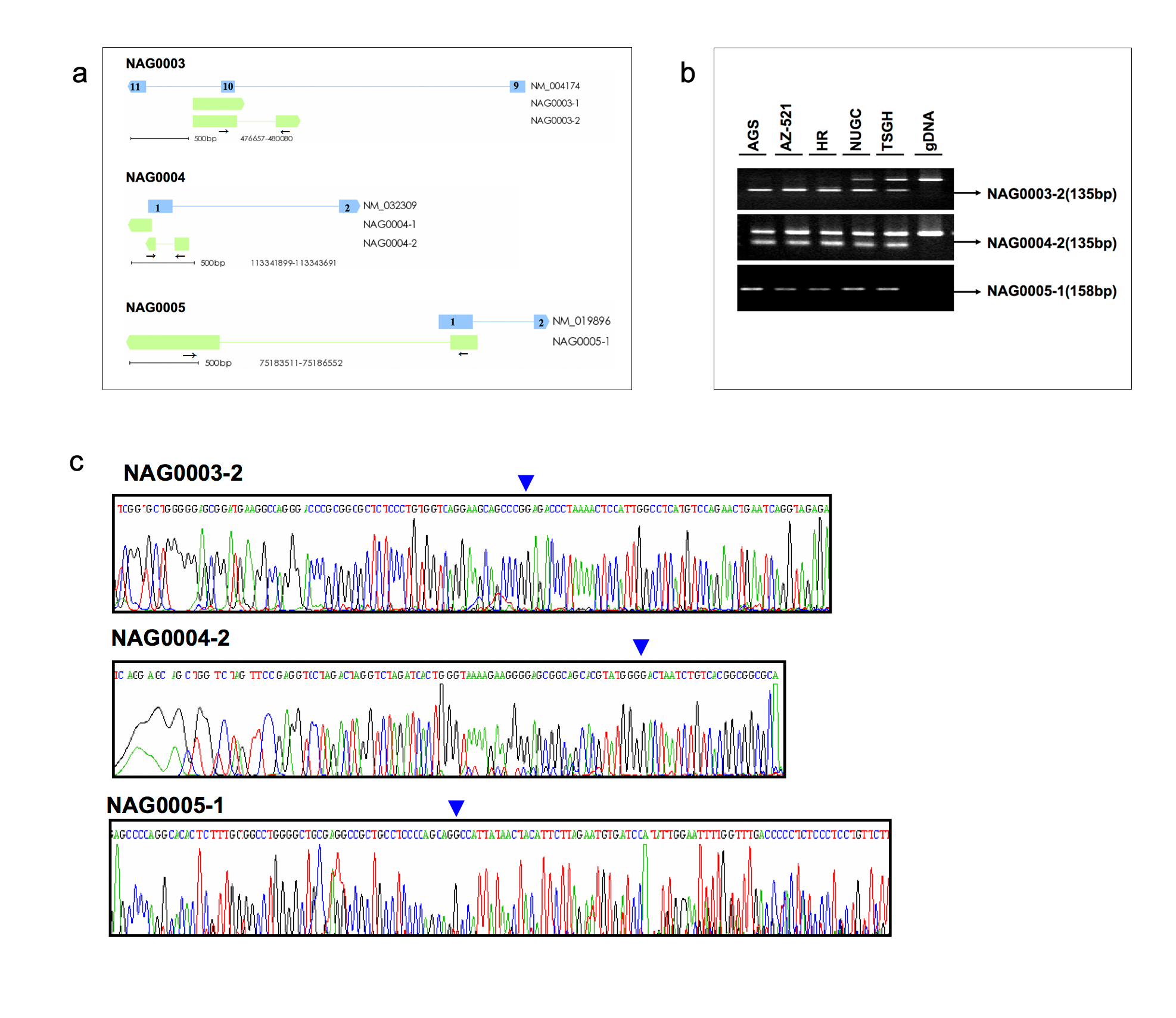


**Supplementary Figure 4. The illustration of novel anti-sense genes overlapped by the same known gene or by the different gene.** The right-forward and left-forward arrows denote plus and minus strands, respectively. The blue and green bars denote the entire genes, rather than the exons as illustrated in Figure 5a. Novel anti-sense gene a, b and c are overlapped by the known Gene A at the anti-sense strand; while, novel anti-sense gene d and e are overlapped by another known Gene B at the anti-sense strand. The fold change values of two novel genes are the ratios of the larger RPKM to the smaller RPKM.


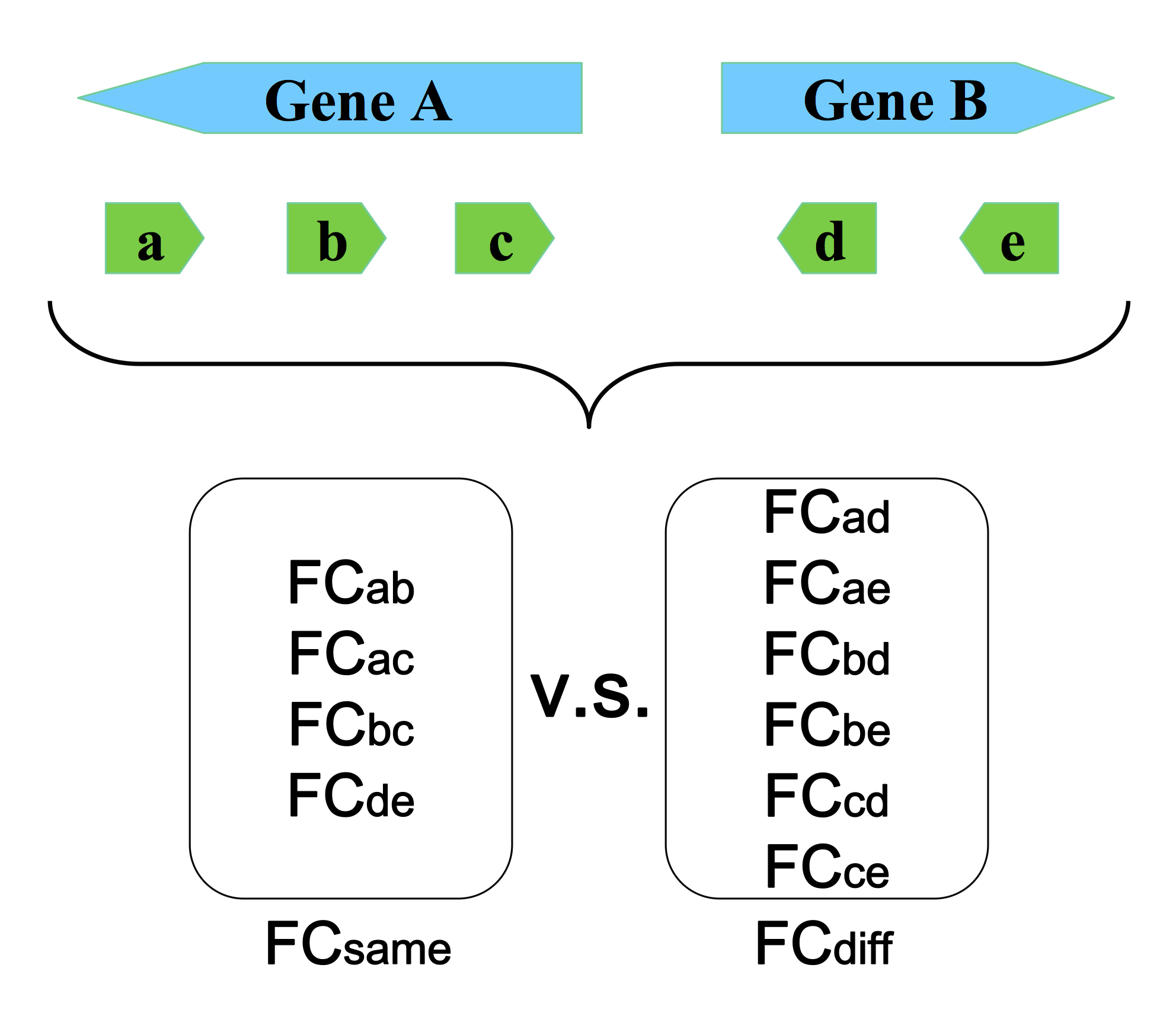


**Supplementary Figure 5. The comparison between FCsame and FCdiff.** The boxplot shows that the values of FCdiff are significantly larger then the ones of FCsame, implicating that the close transcripts could be encoded by the same gene but assembled into two ones owing to the lack of the reads crossing the gap.

**
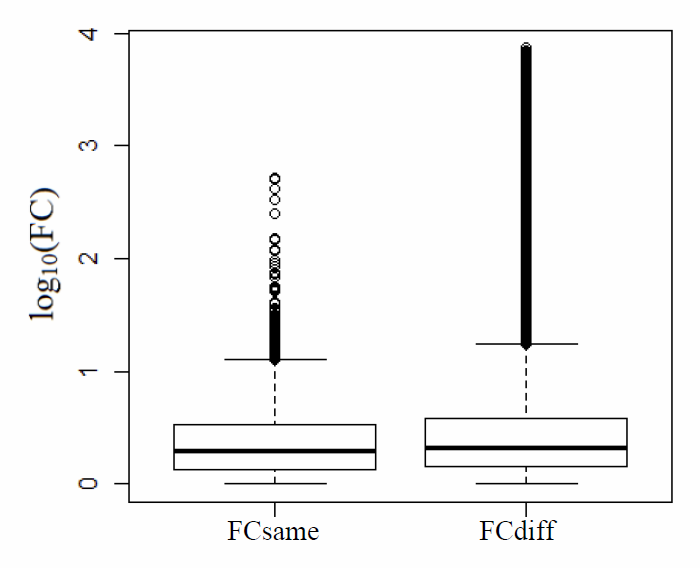
**

**Supplementary Table 1. The PCR and qPCR primers.** The PCR and qPCR primers were designed to avoid the transcripts at the anti-sense strand if applicable.

| **qPCR primers** | |
| --- | --- |
| AMDHD2-F | GATGCCTTCCAGGACTTGCT |
| AMDHD2-R | GAGGTGGGTGATGAAGGTGG |
| ATP6V1C2-F | CTGACTTCAAGGTGGGGACC |
| ATP6V1C2-R | ATGACTTCCACCACGCTCTG |
| COX11-F | GCAGAACAAGACGACCCTCA |
| COX11-R | ACCTGCAACTGCTGATCCTC |
| DALRD3-F | GATCTCCTCTCTGTGCTGGC |
| DALRD3-R | AGCTACTTTCACAGGGCCAC |
| MXD3-F | GAAGCTGGAGGATCAGGAGC |
| MXD3-R | GGGAGTTCCCCCGTTTTCAT |
| POLR2I-F | GGAAGACAAGGAGAACCGCA |
| POLR2I-R | GGACACGTCGGCGATAATCT |
| SDR39U1-F | ACGAAGTGACGTTGGTCTCC |
| SDR39U1-R | CCAGCAATTGGGTGGTCTCT |
| SYNC-F | AGAAGCTGGGACCAAAGCTC |
| SYNC-R | CAGGTTCCTGTTTTGCAGGC |
| TMOD1-F | CGAGGAAAGGTCTGGGTTCC |
| TMOD1-R | TCGCTGCAATGTCACAGAGT |
| EEF1A1-F | CACACGGCTCACATTGCAT |
| EEF1A1-R | CACGAACAGCAAAGCGACC |
| HPRT1-F | GCCAGACTTTGTTGGATTTG |
| HPRT1-R | CTCTCATCTTAGGCTTTGTATTTTG |
| TMEM66-F | AGGAGTCTGGAAAGCAGCAC |
| TMEM66-R | CCGTCACTCAGGAACAGCTT |
| **PCR primers** | |
| NAG0001-1-F | GGTGCTGAGTCAGCCCTQATGT |
| NAG0001-1-R | GGATGAGCGGTGGAGATGACG |
| NAG0002-1-F | ACGGCGAGAAATCAGAGGCCAG |
| NAG0002-1-R | TCGTGTGAGATCCGCGTGCT |
| NAG0003-2-F | GGTGGACGATCGTGGCGTGAAG |
| NAG0003-2-R | CTCTACCTGATTCAGTTCTGGA |
| NAG0004-2-F | GAATCCTGAGGGTCAGATCTCC |
| NAG0004-2-R | GCGCCGCCGTGACAGATTAGTC |
| NAG0005-1-F | CGCTAGCGTCACGTCGGGATCT |
| NAG0005-1-R | GGATCACATTCTAAGAATGTAG |

**Supplementary Table 2. The sets based on different overlap value.** We divided the transcripts into different sets based on the overlap values. The numbers of transcript of sets were also provided.

| **Overlap percentage** | **Correlation coefficient between SS and NSS** | **# transcript** |
| --- | --- | --- |
| O=0 | 0.9635 | 26,873 |
| O>=0.1 | 0.937 | 1,438 |
| O>=0.2 | 0.9331 | 756 |
| O>=0.3 | 0.9326 | 426 |
| O>=0.4 | 0.9138 | 261 |
| O>=0.5 | 0.8933 | 147 |
| O>=0.6 | 0.8605 | 84 |
| O>=0.7 | 0.8435 | 49 |
| O>=0.8 | 0.7758 | 28 |
